# Middle interhemispheric variant of holoprosencephaly in an asymptomatic adult

**DOI:** 10.1259/bjrcr.20190035

**Published:** 2019-11-15

**Authors:** Meltem Özdemir, Aynur Turan, Rasime Pelin Kavak

**Affiliations:** 1 Department of Radiology, University of Health Sciences, Dışkapı Yıldırım Beyazıt Training and Research Hospital, Ankara, Turkey

## Abstract

Middle interhemispheric variant of holoprosencephaly is an uncommon subtype of holoprosencephaly which is characterized by a midline connection of the two cerebral hemispheres in the posterior frontal and parietal regions with the separation of the anterior frontal and occipital lobes. It usually presents in early childhood with facial dysmorphism, seizures, motor dysfunction and mental–motor retardation. We herein present an unusual case of middle interhemispheric variant of holoprosencephaly which was asymptomatic and incidentally found in adulthood.

## Case presentation

A 42-year-old female presenting with a moderate headache of a few weeks was referred to the Department of Radiology for brain MRI. She had neither a neurological disorder nor a cognitive impairment. She had no facial dysmorphism. There was no family history of brain malformations, facial abnormalities, or chromosomal disorders. She was a high school graduate and employed as a civil servant in a government office. She was apparently in a stable marriage and had two healthy children.

## Imaging findings

**Figure 1.**
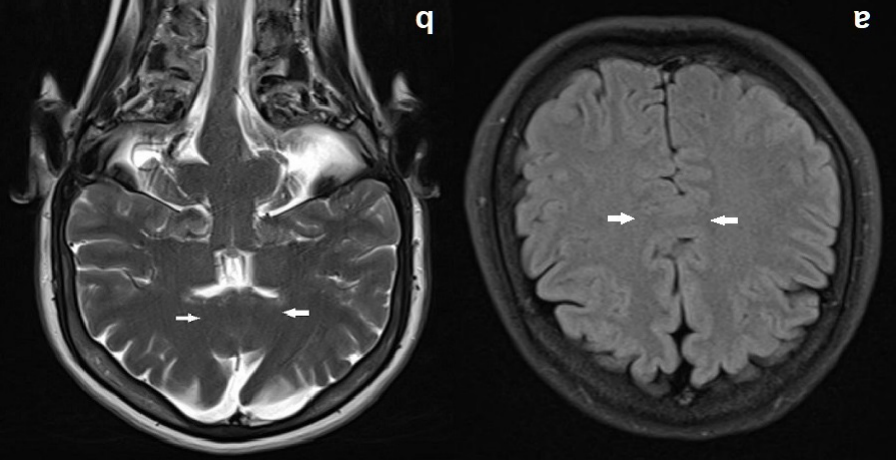
Axial FLAIR (a) and coronal *T*_2_ weighted (b) images show an abnormal interhemispheric fusion in posterior frontal region (white arrows). The interhemispheric fissure and septum pellucidum at the level of the parenchymal connection are absent. Note the thalami and mesencephalon are well separated. FLAIR, fluid-attenuated inversion-recovery.

**Figure 2.**
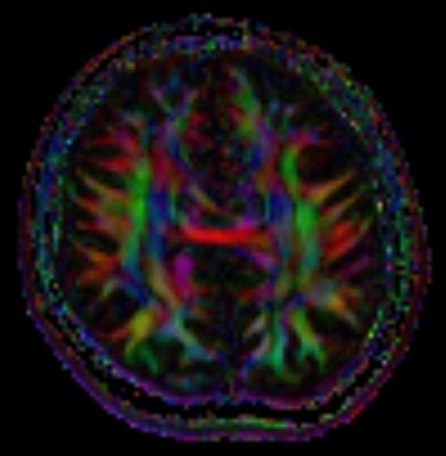
Diffusion tensor imaging section at the level of interhemispheric fusion demonstrates an abnormal transverse white matter tract encoded by red colour. FLAIR, fluid-attenuated inversion-recovery.

**Figure 3.**
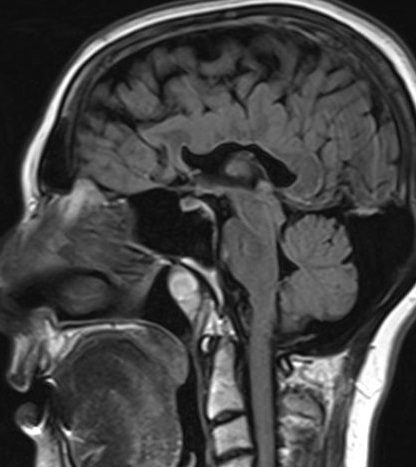
Sagittal FLAIR image shows prominent dysplasia in genu, body and rostral portions of the corpus callosum with a relatively well-formed splenium. Pituitary gland is normal in size and signal intensity. FLAIR, fluid-attenuated inversion-recovery.

**Figure 4.**
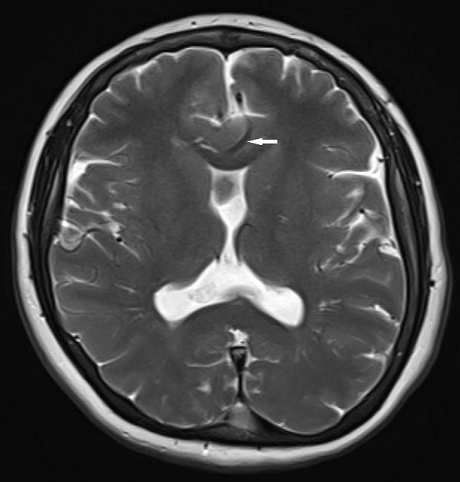
Axial *T*_2_ weighted image at the level of basal ganglia shows normal separation of basal ganglia, thalami, and anterior frontal and occipital lobes. The frontal horns are hypoplastic with absent septum pellucidum. There is an azygos anterior cerebral artery (white arrow).

**Figure 5.**
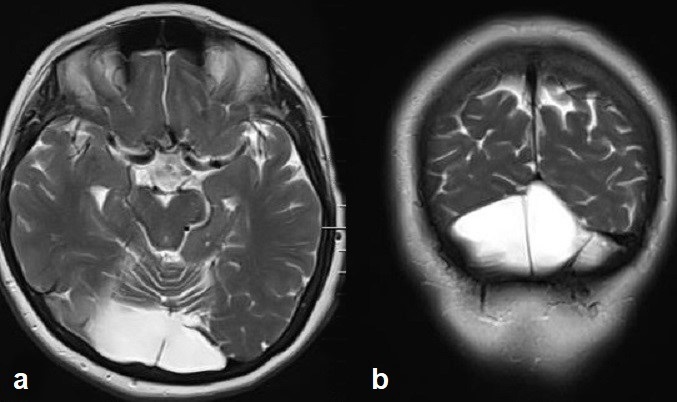
Axial (a) and coronal (a) *T*_2_ weighted images through posterior fossa demonstrate mega-cisterna magna, which may represent a mild form of Dandy–Walker complex.

Brain MRI examination was performed on a 1.5 T MR scanner. Axial and coronal images demonstrated an interhemispheric parenchymal connection and the absence of interhemispheric fissure in the posterior frontal and parietal lobes. Interhemispheric fissure was identifiable in the anterior frontal and occipital lobes (Figure 1). On diffusion tensor imaging (DTI), there was an abnormal transverse white matter tract encoded by red colour at the location of the abnormal interhemispheric fusion (Figure 2). The location, orientation and size of both Sylvian fissures were normal. On sagittal images; all portions of corpus callosum were present with prominently dysplastic genu, body, and rostrum, and a relatively well-formed splenic portion (Figure 3). Both lateral ventricles were of normal width. On axial and coronal images; hypothalamus, caudate nuclei, lentiform nuclei, thalami, and mesencephalon appeared to be normally separated. Pituitary gland, olfactory sulci and bulb were normal in size and configuration. An azygos anterior cerebral artery was recorded (Figure 4). In the posterior fossa, mega-cisterna magna, which may represent a mild form of Dandy-Walker complex, was present (Figure 5). Based on the characteristic imaging findings, the patient was diagnosed as having middle interhemispheric variant of holoprosencephaly (MIVH).

## Treatment

Except for symptomatic medication of headache, no treatment was planned.

## Discussion

MIVH, also termed as “syntelencephaly”, is an uncommon malformation which is characterized by the inadequate separation of the posterior frontal and parietal regions of the two cerebral hemispheres. Although defined as a subtype of holoprosencephaly (HPE), MIVH is different from classic holoprosencephaly in terms of the developmental mechanism, the features of structural abnormalities, and the clinical presentation.^1,2^

HPE is defined as an incomplete separation of the cerebral hemispheres which develops as the result of a defect in the shaping of the basal forebrain during the first month of embryogenesis.^3^ In relation to the severity of the defect, HPE has been classically divided into three types: alobar, semilobar, and lobar. Alobar HPE, the most severe type, appears as a mass of cerebral tissue with a crescent-like monoventricle. In semilobar HPE, posterior falx and posterior interhemispheric fissure are present but anterior brain is non-separated. Lobar HPE is characterized by an inadequate anterior brain separation, well-structured third ventricle and incomplete development of the lateral ventricles.^4^ MIVH, the fourth subtype, is the mildest form with a midline connection at the frontoparietal region.^5^

MIVH was first described by Barkovich and Quint in 1993.^6^ Since then, several subsequent cases and small series describing the structural features and clinical aspects of this rare malformation have been reported. The involvement pattern of MIVH is different from that of classical HPE in which the prosencephalic rostrobasal areas are primarily involved.^7^ Patients with MIVH have a relatively spared basal forebrain with a well-formed anterior interhemispheric fissure. The most anterior portions of the frontal lobes and deep grey nuclei are well-separated in cases with MIVH. Unlike classic HPE, caudate nuclei, lentiform nuclei, and hypothalami are normal or nearly normal with variable fusion of thalami. Sylvan fissures are abnormally connected across the midline in most of the patients with MIVH. While the genu and splenium are relatively well-formed, the body of the corpus callosum is usually deficient at the level of the interhemispheric fusion. The ventricles may show hypoplastic frontal horns with the absence of septum pellucidum. Although most patients have an azygous anterior cerebral artery, cerebral arterial system is relatively spared. The mesencephalon may appear incompletely separated from the diencephalon in up to 18% of patients. A dorsal midline cyst of variable size may be seen. Heterotopic grey matter, cortical dysplasia, polymicrogyria, and the posterior fossa abnormalities may accompany MIVH.^1,8–10^

Most patients with MIVH have mild-to-moderate facial dysmorphism including hypertelorism, cleft lip and palate, and are usually diagnosed prenatally or in early childhood. The most common clinical findings seen in the affected children are; seizures, motor dysfunctions, and mental-motor retardation. In a recent study with 15 patients having MIVH; 40% of patients had a history of at least one seizure, 86% had mild-to-moderate spasticity, 57% had hypotonia of various degrees, and 50% had dystonia.^2^ Speech and oromotor development were delayed in all patients. Endocrinopathies, choreoathetosis, or severe midline craniofacial anomalies which are commonly observed in other forms of HPE were not present in any of the 15 patients. To the best of our knowledge, the present case is the first case of MIVH reported to be asymptomatic and found incidentally in adulthood. Recently, an adult MIVH case without any neurological disorder but with neuropsychological abnormalities that has been observed since childhood has been reported.^11^ While the mentioned case was reported to have experienced learning disability and memory related functional deterioration since childhood, our case did not report any serious problems regarding her education and she graduated from high school in the same period with her peers. These two cases raise the question whether the mild forms of MIVH are more prevalent in population than they have been expected to be. If so, how and why the characteristic morphologic abnormalities of MIVH cause specific clinical disorders in some individuals whereas they seem to stay asymptomatic in some others, are the questions to be answered.

ZIC2 gene which is located on human chromosome 13q32, is one of the four genes accused of causing human HPE.^12^ In a recent study with 509 cases of HPE, a ZIC2 mutation was noted in 16 cases. Among these 16 cases; 15 had alobar, semilobar, or lobar HPE. In the remaining one who had MIVH, a comparatively mild mutation of the ZIC2 gene (in-frame deletion of 12 amino acids) was observed.^13^ This finding suggests that the severity of the malformation, ranging from MIVH to alobar HPE, might depend on the level of the function of the protein of which the structure is damaged.^8^

Advances in the imaging techniques and wide availibility of advanced diagnostic modalities allow accurate diagnosis of both the severe and the mild forms of HPE. Further investigations are needed to correlate the characteristic morphologic abnormalities of the disorder with its specific clinical outcomes, and to prognosticate the developmental potential of affected individuals.

## Learning points

1. MIVH is a rare midline malformation in which the cerebral hemispheres fail to divide in the posterior frontal and parietal regions.
2. MIVH usually presents in early childhood with facial dysmorphism and neorological disturbances.
3. MIVH may remain asymptomatic.

## References

[1] Simon EM, Hevner RF, Pinter JD, Clegg NJ, Delgado M, Kinsman SL, et al The middle interhemispheric variant of holoprosencephaly. AJNR Am J Neuroradiol 2002; 23: 151–6. 11827888 PMC7975493

[2] Lewis AJ, Simon EM, Barkovich AJ, Clegg NJ, Delgado MR, Levey E, et al Middle interhemispheric variant of holoprosencephaly: a distinct cliniconeuroradiologic subtype. Neurology 2002; 59: 1860–5. doi: 10.1212/01.WNL.0000037483.31989.B9 12499474

[3] Golden JA Towards a greater understanding of the pathogenesis of holoprosencephaly. Brain Dev 1999; 21: 513–21. doi: 10.1016/S0387-7604(99)00067-4 10598051

[4] Barkovich A. J Pediatric Neuroimaging, 3rd ed Philadelphia: Lippincott Williams & Wilkins; 2000 .

[5] Simon EM, Barkovich AJ Holoprosencephaly: new concepts. Magn Reson Imaging Clin N Am 2001; 9: 149–64. 11278187

[6] Barkovich AJ, Quint DJ Middle interhemispheric fusion: an unusual variant of holoprosencephaly. AJNR Am Neuroradiol 1993; 14: 431–40. PMC8332955 8456724

[7] Simon EM, Hevner R, Pinter JD, Clegg NJ, Miller VS, Kinsman SL, Hahn J, Barkovich AJ, et al Assessment of the deep gray nuclei in holoprosencephaly. AJNR Am J Neuroradiol 2000; 21: 1955–61. 11110554 PMC7974288

[8] Takanashi J-I, Barkovich AJ, Clegg NJ, Delgado MR Middle interhemispheric variant of holoprosencephaly associated with diffuse polymicrogyria. AJNR Am J Neuroradiol 2003; 24: 394–7. 12637288 PMC7973611

[9] Pulitzer SB, Simon EM, Crombleholme TM, Golden JA Prenatal MR findings of the middle interhemispheric variant of holoprosencephaly. AJNR Am J Neuroradiol 2004; 25: 1034–6. 15205143 PMC7975649

[10] Atalar MH, Icagasioglu D, Sener RN Middle interhemispheric variant of holoprosencephaly associated with bilateral perisylvian polymicrogyria. Pediatr Int 2008; 50: 241–4. doi: 10.1111/j.1442-200X.2007.02311.x 18353069

[11] Virta M, Launes J, Valanne L, Hokkanen L Adult with Middle Interhemispheric Variant of Holoprosencephaly: Neuropsychological, Clinical, and Radiological Findings. Arch Clin Neuropsychol 2016; 31: 472–9. doi: 10.1093/arclin/acw026 27235161

[12] Marcorelles P, Loget P, Fallet-Bianco C, Roume J, Encha-Razavi F, Delezoide A-L Unusual variant of holoprosencephaly in monosomy 13q. Pediatr Dev Pathol 2002; 5: 170–8. doi: 10.1007/s10024001-0200-5 11910512

[13] Brown LY, Odent S, David V, Blayau M, Dubourg C, Apacik C, et al Holoprosencephaly due to mutations in ZIC2: alanine tract expansion mutations may be caused by parental somatic recombination. Hum Mol Genet 2001; 10: 791–6. doi: 10.1093/hmg/10.8.791 11285244

